# Correction: Prina et al. Relationship between Environmental Conditions and Utilisation of Community-Based Mental Health Care: A Comparative Study before and during the COVID-19 Pandemic in Italy. *Int. J. Environ. Res. Public Health* 2024, *21*, 661

**DOI:** 10.3390/ijerph22060898

**Published:** 2025-06-05

**Authors:** Eleonora Prina, Federico Tedeschi, Antonio Lasalvia, Damiano Salazzari, Sara Latini, Laura Rabbi, Federica Marando, Elaine van Rijn, Jan Wollgast, Enrico Pisoni, Bertrand Bessagnet, Maxime Beauchamp, Francesco Amaddeo

**Affiliations:** 1 Department of Neurosciences, Biomedicine and Movement Science, Section of Psychiatry, University of Verona, 37134 Verona, Italy; federico.tedeschi@univr.it (F.T.); antonio.lasalvia@univr.it (A.L.); salazzari@gmail.com (D.S.); sara.latini@univr.it (S.L.); laura.rabbi@univr.it (L.R.); francesco.amaddeo@univr.it (F.A.); 2 European Commission, Joint Research Centre (JRC), 21027 Ispra, Italy; federica.marando@ec.europa.eu (F.M.); elaine.van-rijn@ec.europa.eu (E.v.R.); jan.wollgast@ec.europa.eu (J.W.); enrico.pisoni@ec.europa.eu (E.P.); bertrand.bessagnet@ec.europa.eu (B.B.); 3 IMT Atlantique, 655 Av. du Technopôle, 29280 Plouzané, France; maxime.beauchamp76@gmail.com

In the original publication [1], there was an error regarding the affiliation for Maxime Beauchamp. In addition to affiliations 1,2, the updated affiliation should include 3 IMT Atlantique, 655 Av. du Technopôle, 29280 Plouzané, France. The authors state that the scientific conclusions are unaffected. This correction was approved by the Academic Editor. The original publication has also been updated.
