# Supplementary material for: Relationship between Environmental Conditions and Utilisation of Community-Based Mental Health Care: A Comparative Study before and during the COVID-19 Pandemic in Italy
Source: Int J Environ Res Public Health. 2024 May 22;21(6):661. doi: 10.3390/ijerph21060661 (PMC11203518; doi:10.3390/ijerph21060661)
Supplement: Supplementary file 1 [file ijerph-21-00661-s001.zip › ijerph-2980920-supplementary(3)(4).pdf]

International Journal of Environmental Research and Public Health

Supplementary Materials

Title: **Relationship between Environmental Conditions and Utilisation of Community-Based Mental Health Care: A Comparative Study before and during the COVID-19 Pandemic in Italy**

INDEX

Supplementary File S1: STROBE checklist (p. 3)

Supplementary File S2: P-value of tests involving multiple regression parameters (p. 5)

Supplementary File S3: Calculation of the environmental and socio-demographic variables (p. 6)

Supplementary File S1

STROBE Statement—checklist of items that should be included in reports of observational studies

	Item No	Recommendation	Page No
**Title and abstract**	1	(*a*) Indicate the study's design with a commonly used term in the title or the abstract	0 - 1
		(*b*) Provide in the abstract an informative and balanced summary of what was done and what was found	1
**Introduction**			
Background/rationale	2	Explain the scientific background and rationale for the investigation being reported	2 - 4
Objectives	3	State specific objectives, including any prespecified hypotheses	4
**Methods**			
Study design	4	Present key elements of study design early in the paper	4
Setting	5	Describe the setting, locations, and relevant dates, including periods of recruitment, exposure, follow-up, and data collection	4 - 7
Participants	6	(*a*) *Cohort study*—Give the eligibility criteria, and the sources and methods of selection of participants. Describe methods of follow-up<br>*Case-control study*—Give the eligibility criteria, and the sources and methods of case ascertainment and control selection. Give the rationale for the choice of cases and controls<br>*Cross-sectional study*—Give the eligibility criteria, and the sources and methods of selection of participants	4 - 7
		(*b*) *Cohort study*—For matched studies, give matching criteria and number of exposed and unexposed<br>*Case-control study*—For matched studies, give matching criteria and the number of controls per case	4 - 7
Variables	7	Clearly define all outcomes, exposures, predictors, potential confounders, and effect modifiers. Give diagnostic criteria, if applicable	4 - 7
Data sources/ measurement	8*	For each variable of interest, give sources of data and details of methods of assessment (measurement). Describe comparability of assessment methods if there is more than one group	6 - 7
Bias	9	Describe any efforts to address potential sources of bias	12
Study size	10	Explain how the study size was arrived at	5
Quantitative variables	11	Explain how quantitative variables were handled in the analyses. If applicable, describe which groupings were chosen and why	7 - 9
Statistical methods	12	(*a*) Describe all statistical methods, including those used to control for confounding	7 - 9
		(*b*) Describe any methods used to examine subgroups and interactions	7 - 9
		(*c*) Explain how missing data were addressed	7 - 9
		(*d*) *Cohort study*—If applicable, explain how loss to follow-up was addressed<br>*Case-control study*—If applicable, explain how matching of cases and controls was addressed	NA

		Cross-sectional study—If applicable, describe analytical methods taking account of sampling strategy	7
		(*e*) Describe any sensitivity analyses	NA

Results

Participants	13*	(a) Report numbers of individuals at each stage of study—eg numbers potentially eligible, examined for eligibility, confirmed eligible, included in the study, completing follow-up, and analysed	9, Table 1
		(b) Give reasons for non-participation at each stage	9
		(c) Consider use of a flow diagram	NA
Descriptive data	14*	(a) Give characteristics of study participants (eg demographic, clinical, social) and information on exposures and potential confounders	9, Table 1
		(b) Indicate number of participants with missing data for each variable of interest	Tables
		(c) *Cohort study*—Summarise follow-up time (eg, average and total amount)	NA
Outcome data	15*	*Cohort study*—Report numbers of outcome events or summary measures over time	
		Case-control study—Report numbers in each exposure category, or summary measures of exposure	
		Cross-sectional study—Report numbers of outcome events or summary measures	*9 - 10*
Main results	16	(*a*) Give unadjusted estimates and, if applicable, confounder-adjusted estimates and their precision (eg, 95% confidence interval). Make clear which confounders were adjusted for and why they were included	9 - 10
		(*b*) Report category boundaries when continuous variables were categorized	Tables
		(*c*) If relevant, consider translating estimates of relative risk into absolute risk for a meaningful time period	9 - 10
Other analyses	17	Report other analyses done—eg analyses of subgroups and interactions, and sensitivity analyses	Appendix

Discussion

Key results	18	Summarise key results with reference to study objectives	10
Limitations	19	Discuss limitations of the study, taking into account sources of potential bias or imprecision. Discuss both direction and magnitude of any potential bias	12
Interpretation	20	Give a cautious overall interpretation of results considering objectives, limitations, multiplicity of analyses, results from similar studies, and other relevant evidence	11 - 12
Generalisability	21	Discuss the generalisability (external validity) of the study results	11 - 12

Other information

Funding	22	Give the source of funding and the role of the funders for the present study and, if applicable, for the original study on which the present article is based	NA

*Give information separately for cases and controls in case-control studies and, if applicable, for exposed and unexposed groups in cohort and cross-sectional studies.

Note: An Explanation and Elaboration article discusses each checklist item and gives methodological background and published examples of transparent reporting. The STROBE checklist is best used in conjunction with this article (freely available on the Web sites of PLoS Medicine at http://www.plosmedicine.org/, Annals of Internal Medicine at

http://www.annals.org/, and Epidemiology at http://www.epidem.com/). Information on the STROBE Initiative is available at www.strobe-statement.org.

Supplementary File S2

P-values of global tests on regression parameters

Regression parameters	Test statistics	P-value
Model 1:<br>all sociodemographic variables	$X^2(3) = 15.00$	0.002
Model 1:<br>all environmental variables	$X^2(9) = 40.57$	<0.001
Model 2:<br>all interactions	$X^2(12) = 138.23$	<0.001
Model 2:<br>interactions sociodemographic variables	$X^2(3) = 90.85$	<0.001
Model 2:<br>interactions environmental variables	$X^2(9) = 31.49$	<0.001
Model 2:<br>all parameters on sociodemographic variables	$X^2(6) = 105.91$	<0.001
Model 2:<br>all parameters on environmental variables	$X^2(18) = 73.01$	<0.001
Model 3:<br>all environmental variables	$X^2(9) = 7.06$	0.630
Model 3:<br>all sociodemographic variables	$X^2(3) = 15.13$	0.002
Model 3:<br>parameters related to COVID-19 restrictions	$X^2(2) = 28.50$	< 0.001

Supplementary File S3

Calculation of the environmental and socio-demographic variables

Census block centroids

To better estimate population exposure to the environmental variables, centroids of each census block, approximating where the majority of the population in each census block resides, were estimated. In order to do so, the weighted mean distance of all the residential buildings in each census block was used to estimate the position of the centroids. Data for the residential building was retrieved from Copernicus Urban Atlas Land Cover/Land Use data (year 2018) (Copernicus, 2023a) [1]. Census block geometries were retrieved from the ISTAT (Italian Statistical Office) website (Istat, 2023) [2].

Solar Radiation

Solar Radiation (in W m^{-2}) was retrieved from Copernicus ECMWF ERA5 reanalysis for the global climate and weather (name of the variable: surface solar radiation downwards). It is the solar radiation reaching the surface of the earth, and it comprise both direct and diffuse solar radiation. The total daily accumulated value of solar radiation for each day for the years 2019, 2020 and 2021 was selected and downloaded. Then, in order to transform the J m^{-2} to W m^{-2}, the values were divided by the accumulation period in seconds. Finally, total weekly values were computed and averaged by census block.

Percentage of trees in a Census Block

Data on tree canopy cover, at a spatial resolution of 10 m, was retrieved from Copernicus High Resolution Layer tree cover density layer for the year 2018 (Copernicus, 2023b) [3]. Then, a circular buffer of 300 m of radius from the centroid was produced, and the percentage of trees within each 300 m buffer, per census block, was estimated.

Share of green areas in a Census Block

The total amount of green areas in the province of Verona, both public and private, was estimated through the methodology applied in Zulian et al., 2022 [4] for the variable named "greenness", defined as "the amount of vegetation present in urbanized areas". The share of green areas in each census block was calculated by computing the total area of greenness inside each circular buffer of 300 m of radius around the census block centroids.

Presence of watercourses and of large public green areas within 300 meters from the Census Block centroid

All the large public green areas (with an area above 2 hectares) and water courses in the city of Verona were selected from different spatial datasets: the Copernicus Urban Atlas Land Cover/Land Use data (year 2018) [1], OpenStreetMap, and data from the Municipality of Verona administration. Then, the minimum distance from all the large public green areas and water courses and the census block centroids was computed. If the centroid was within a distance of 300 m, a value of 1 was assigned, if the distance was higher, then a value of 0 was assigned, producing a dichotomous variable expressing the proximity of each census block to green and blue areas.

Socio-demographic variables

The data for calculating the socio-demographic variables (low- schooling index, unemployment rate and the percentage of households living in rented accommodation) was retrieved from the ISTAT census registry (year 2011). The indexes were calculated for each census block.

References

1. Copernicus. Urban Atlas Land Cover/Land Use 2018 (vector), Europe, 6-yearly. 2023. Available online: https://land.copernicus.eu/en/products/urban-atlas/urban-atlas-2018 (accessed on 2 November 2023).

2. Istat. Basi territoriali e variabili censuarie. **2023**. Available online: https://www.istat.it/it/archivio/104317 (accessed on 2 November 2023).

3. Copernicus. Tree Cover Density 2018 (raster 10 m and 100 m), Europe, 3-yearly. 2023. Available online: https://land.copernicus.eu/en/products/high-resolution-layer-tree-cover-density/tree-cover-density-2018 (accessed on 2 November 2023).

4. Zulian, G.; Marando, F.; Mentaschi, L.; Alzetta, C.; Wilk, B.; Maes J. Green balance in urban areas as an indicator for policy support: a multi-level application. *One Ecosyst.* **2022**, *7*, 1–39.
